# CBD Disrupts Malme-3M Cell Metabolism via Glycolytic Shift and Redox Imbalance

**DOI:** 10.3390/cimb47110928

**Published:** 2025-11-06

**Authors:** Laura M. Shelton, Yiling Xu, Hans Ghayee, Alexander M. Buko

**Affiliations:** 1 bitBiome, Inc., Shinjuku 169-0051, Japan; 2 Department of Medicine, University of Florida, 1600 SW Archer Rd., Gainesville, FL 32608, USA; xuyiling870@gmail.com (Y.X.); hans.ghayee@medicine.ufl.edu (H.G.); 3 Human Metabolome Technologies, America, 24 Denby Road, Suite 217, Boston, MA 02134, USA; alex.buko@humanmetabolome.com

**Keywords:** Malme-3M, BJ fibroblast, cannabidiol (CBD), redox, metabolism

## Abstract

Background: Accumulating evidence suggests that cannabidiol (CBD) exerts variable effects on cancer cells that influence cellular activity, including growth. While anecdotal evidence abounds, mechanistic studies have lagged. Methods: Malme-3M cells derived from melanoma and less-aggressive BJ fibroblast cells were incubated with CBD. CE-MS mass spectroscopy was used to measure metabolite changes resulting from CBD treatment. Results: Data indicate a differential response between malignant Malme-3M cells and BJ fibroblasts with respect to metabolites critical for primary metabolic function. A significant reduction in TCA metabolites is seen with a corresponding increase in glycolytic output in the Malme-3M cell line. A similar reduction in TCA activity in BJ fibroblasts appears to differentially activate fatty acid oxidation. ATP is significantly reduced in the Malme-3M cells with a corresponding decrease in metabolites associated with redox maintenance. Conclusions: This is the first metabolomics analyses of malignant Malme-3M cells and less-aggressive BJ fibroblasts after pre-treatment with CBD. The data suggest that the CBD-induced metabolic perturbation could reprogram cellular metabolism and affect ATP production and redox maintenance of the more-aggressive Malme-3M cells.

## 1. Introduction

In recent years, the medical and scientific communities have witnessed a surge of interest in the potential therapeutic applications of cannabidiol (CBD), a non-psychoactive compound derived from the *Cannabis sativa* plant. Of the over 500 compounds in the *C. sativa* plant, 60 have been classified as cannabinoids (CBs) with 2, Delta-9-tetrahydrocannabinol (THC) and Cannabidiol (CBD), having been extensively tested in a research setting. However, interest has increased in the minor compounds found in the flower as well as the rest of the plant [1].

CBs have been experimented on as part of treatments for chemotherapy–toxicity-induced indications. CBs are effective in reducing nausea associated with chemotherapy and radiotherapy. However, CBs have also been shown to have anti-proliferative effects on several cancers including breast, colorectal, lung, paraganglioma, liver, melanoma, and brain, both in vitro and in vivo [2,3,4,5,6,7]. Evidence suggests that CBD may exert anticancer effects through multiple mechanisms, including apoptosis induction, cell cycle regulation, inhibition of angiogenesis, and attenuation of migration as well as invasion [2,8].

The effects of CBs are due in part to signaling through endogenous cannabinoid receptors (CB-Rs). These receptors are present on several cancer cell lines and directly correlate with aggressiveness [7,9]. These effects have been shown on both estrogen positive and negative breast cancer cell lines. In addition, effects have been seen in models of gliomas and neuroblastomas where Phase 1 clinical trials of combination temozolomide and CBD for the treatment of highly invasive brain cancer have shown promising results [1,10,11]. Interest lies not only in the correlation of CB-Rs with aggressiveness but also in the receptor activation of known cancer-inducing pathways, including the PI3K-AKT pathway [12]. CBD has also been found to have therapeutic potential in a model of gemcitabine-resistant cholangiocarcinoma, offering new potential therapies to drug-resistant cases [13]. Interestingly, recent evidence even shows a positive correlation among CBD usage and inhibition of viral replication of SARS-CoV-2 infections [11].

However, CB’s effect on cancer cell metabolism has yet to be examined. Cancer cell metabolism has been a heavily researched topic, often attributed to Otto Warburg’s work. Warburg postulated that all cancers suffered from an irreversible inhibition of mitochondrial metabolism, leaving cancer cells wholly dependent on glycolysis and sugar for energy production [14]. In recent years, however, we have seen the complexity of cancer cell metabolism; studies show that glutamine and fatty acids also play a role in cancer growth and survival. Glutamine plays a key role in the regulation and maintenance of redox potential as it is a key precursor for glutathione synthesis. However, this also relies heavily on glucose metabolism via the pentose phosphate pathway (PPP), which is responsible for the production of the reducing equivalent NADPH, critical for the recycling of oxidized glutathione (GSSSG) back to its reduced form (GSH) [15,16,17]. However, targeting cancer cell metabolism has remained an interesting yet elusive target for novel therapies. It has been suggested that CBs may exert their anti-proliferative effects partly through changes in mitochondrial membrane potential, suggesting a metabolic consequence of treatment with CBs [18].

Aside from their use in cancer therapy, CBs have been shown to influence pain and inflammation through their modulation of antioxidant enzymes such as superoxide dismutase and thioredoxin reductase, as well as its modulation of the inflammatory marker NFkB [19,20]. Application of CBs therefore reach wide from cancer to Parkinson’s, and even to chronic pain [21,22]. Furthermore, CBD has been shown to modulate several neuropsychiatric disorders with the potential to modulate psychotic, addictive, and depressive states [23].

Currently, the US Food and Drug Administration (FDA) has not approved any cannabis products for the treatment of diseases. However, it has approved several cannabis-derived and synthetic THC-like compounds for the treatment of seizures and AIDS-associated weight loss (Epidolex, Marinol, Syndros, and Cesamet). Research on large human cohorts is limited partly because important metabolic studies are lacking.

In this study, our aim was to understand the metabolic changes in malignant Malme-3M cells compared to less-aggressive BJ fibroblasts after treatment with CBD.

## 2. Materials and Methods

### 2.1. Cell Culture

The Malme-3M, (ATCC Catalog No. HTB-64, Manassas, VA, USA), were grown in an IMDM medium (Gibco, Catalog No.12440-053, Waltham, MA, USA) with 10% FBS. Immortalized BJ fibroblasts, gift from Jerry Shay at UT Southwestern (Dallas, TX, USA), were grown in a DMEM medium (Gibco, Catalog No. 11965-092, Waltham, MA, USA) with 10% FBS.

Cells were grown in T75 flasks until they reached logarithmic phase growth (80% confluency), then the cells were seeded into four replicate 60 mm dishes. The seeding density of BJ cells was 3 × 10^5^ cells/dish and for Malme-3M was 1.2 × 10^6^ cells/dish. The cells were incubated for 24 h before treatment. Cells were then treated with DMSO (vehicle, Sigma Catalog No. D2650-100ML, Burlington, MA, USA) or CBD (Cayman Chemical Catalog No. 90080, Ann Arbor, MI, USA) and incubated at 37 °C in a CO_2_ incubator for 72 h before metabolite extraction.

### 2.2. Dosing with CBD

BJ fibroblasts were dosed with 12.5 µM CBD, and Malme-3M cells were dosed with 16 µM CBD for 72 h, which represent their respective IC50 values as determined by cell viability assays at varying concentrations of CBD, as shown in Supplementary Figure S1.

### 2.3. Metabolite Measurements

Triplicate 60 mm dishes of cultured cells were used for the extraction of intracellular metabolites. The culture medium was aspirated from the dish and cells were washed twice by 5% mannitol solution (5 mL first and then 1 mL). The cells were then treated with 400 µL of methanol and left at rest for 30 s to inactivate enzymes. Next, the cell extract was treated with 275 µL of Milli-Q water containing internal standards (H3304-1002, Human Metabolome Technologies, Inc., Tsuruoka, Japan) and left at rest for another 30 s. The extract was obtained and centrifuged at 2300× *g* and 4 °C for 5 min and then 350 µL of the upper aqueous layer was centrifugally filtered through a Millipore 5 kDa cutoff filter at 9100× *g* and 4 °C for 180 min to remove proteins. The filtrate was centrifugally concentrated and re-suspended in 50 µL of Milli-Q water for CE-MS analysis.

The untargeted metabolome analysis with CE-TOF/MS was performed using an Agilent CE-TOFMS system (Agilent Technologies, Santa Clara, CA, USA) equipped with a fused silica capillary [50 µm (inner diameter) × 80 cm]. For measurements of cationic/anionic metabolites, running buffer, a solution composed of Cation Buffer Solution [H3301–1001; Human Metabolome Technologies (HMT), Tsuruoka, Japan] and Anion buffer solution (H3302–1021) were used, with CE voltage + 27 kV/+ 30 kV, MS ionization ESI positive/negative, MS capillary voltage 4000 V/3500 V, MS scan range 50–1000 m/z, and HMT Sheath Liquid (H3301–1020). Identification of metabolites and measurement of relative amounts were performed using MASTER HANDS (version 2.1.0.1, 2.9.0.9; Keio University, Tokyo, Japan) and the HMT metabolite database based on internal standards (HMT) [24]. Data are represented as the average of three independent samples +/− standard deviation. All metabolite measurements are represented as the relative area under the curve.

## 3. Results

In this study, we aimed to understand metabolic changes in the malignant Malme-3M cell line compared to the less-aggressive BJ fibroblast cell line when pre-treated with cannabidiol (CBD) for 72 h. We first interrogated cellular wide changes and their associated metabolic processes. BJ fibroblast and Malme-3M cell lines were treated with 12.5 and 16 μM CBD, which, respectively, represent the IC50 for each cell line. As shown in Table 1, we saw a larger number of differentially regulated metabolites in the Malme-3M cell line treated with CBD relative to BJ fibroblasts. Of note, we saw significant changes in several key metabolic pathways to include glycolysis, glutaminolysis, redox maintenance, the urea cycle, and the TCA cycle.

**Figure 1:**
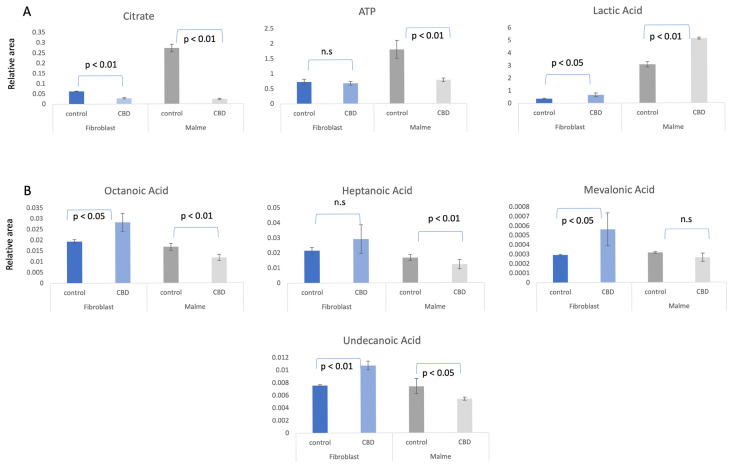
(**A**). Primary metabolic response to CBD treatment. BJ Fibroblast and Malme-3M cell lines were treated with 12.5 and 16 μM CBD for 72 h, which, respectively, represent the IC50 for each cell line. Metabolite measurements are represented as the average relative area under the curve of 3 independent replicates per measurement. While the fibroblast cell line showed a significant decrease in the TCA cycle metabolite citrate, there was no corresponding increase in glycolytic activity as shown by no significant difference in ATP concentrations. However, with the marked reduction in citrate levels in the Malme cell line, there was a corresponding significant decrease in ATP. Glycolytic activity was correspondingly increased in the Malme cell line in a likely attempt to maintain ATP levels. (**B**). Fatty acid metabolism is elevated in fibroblasts cells because of CBD treatment. Short chain fatty acids are found to be elevated in the fibroblast cell line in response to CBD treatment with significant changes seen in octanoic, mevalonic, and undecanoic acid. In contrast, there is an overall slight decrease or no change in the Malme cell line, consistent with an inhibited TCA cycle. *p* values represent the standard deviation of *n* = 3 for all samples, n.s. denotes not significant.

While both the less-aggressive BJ fibroblast cells and aggressive Malme-3M cells showed a significant decrease in citrate, as shown in Figure 1A, there was only a corresponding decrease in ATP in the Malme-3M cells. Glycolytic activity was correspondingly increased in the Malme-3M cell line in a likely attempt to maintain ATP levels, as shown by a significant increase in lactic acid production.

By examining the change in short and medium chain fatty acids, we observed that fatty acid metabolism is elevated in BJ fibroblasts because of CBD treatment, as shown in Figure 1B. This suggests that the BJ fibroblasts respond to reductions in TCA cycle activity with an increase in fatty acid oxidation to maintain constant ATP levels. In contrast, there is an overall significant decrease or no change in the Malme-3M cells, consistent with an inhibited TCA cycle.

**Figure 2:**
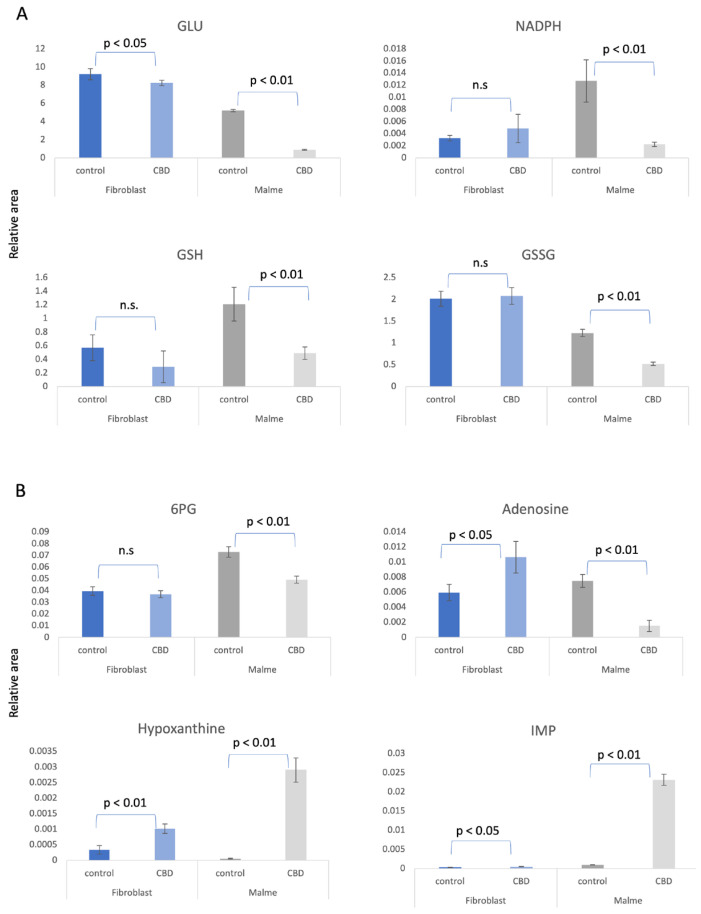
(**A**) Treatment for 72 h with CBD results in potential oxidative stress or greater susceptibility to oxidative stress in malignant Malme-3M cells only. Glutamate (GLU) was significantly reduced in Malme-3M cells, resulting in a significant reduction in both oxidized and reduced glutathione (GSSG, GSH, respectively), likely affecting de novo glutathione synthesis. Nicotinamide adenine dinucleotide phosphate (NADPH), which is required for GSH regeneration, was also significantly reduced in Malme-3M only. The pentose phosphate pathway and de novo purine synthesis is differentially regulated by CBD in BJ fibroblasts vs. Malme-3M cells. (**B**) As a result of a likely increase in glycolytic activity in the Malme-3M cells, glucose carbons are shunted away from the PPP to accommodate this increased demand for ATP, as shown by a significant decrease in 6PG, the first metabolite of the PPP. This results in an overall decrease in de novo purine synthesis, as shown in a dramatic decrease in adenosine levels. The nucleotide salvage pathway can potentially recycle purine bases to maintain some level of de novo purine synthesis. We see hypoxanthine and IMP, a product recycled from hypoxanthine, is elevated in Malme-3M + CBD, suggesting nucleotide salvage is activated in the Malme-3M cells. In contrast, we see elevations of all the purine metabolites in the BJ fibroblasts, suggesting de novo purine synthesis may be slightly elevated. Metabolite measurements are represented as the average relative area under the curve of 3 independent replicates per measurement. *p* values represent the standard deviation of *n* = 3 for all samples, n.s., denotes not significant.

We suggest that CBD results in potential oxidative stress—or greater susceptibility to oxidative stress—in malignant cells lines only. In Figure 2A, we show that glutamate was significantly reduced in the Malme-3M cells, resulting in a reduction in both GSH and GSSG, likely affecting de novo glutathione synthesis. NADPH, which is required for GSH regeneration, was also significantly reduced in Malme-3M.

As shown in Figure 2B, the pentose phosphate pathway (PPP) and de novo purine synthesis are differentially regulated by CBD in BJ fibroblasts versus the Malme-3M cells. As a result of a likely increase in glycolytic activity in the Malme-3M cell line, glucose carbons are shunted away from the PPP to accommodate this increased demand for ATP. This results in an overall decrease in de novo purine synthesis, as shown by a dramatic decrease in both 6-phosphogluconate (6-PG), the first metabolite of the PPP, and adenosine levels, a key precursor for nucleotide synthesis. The nucleotide salvage pathway can potentially recycle purine bases to maintain some level of de novo purine synthesis. During this process of recycling nucleotides, hypoxanthine is a key intermediate. We see both hypoxanthine and inosine monophosphate (IMP), a product recycled from hypoxanthine, are dramatically elevated in Malme-3M + CBD with a 53× and 23× fold increase, respectively, suggesting nucleotide salvage is activated. The first committed step of the PPP also produces NADPH, which is essential for GSH regeneration. We see that in Malme-3M, this step seems to be reduced, resulting from less activity in the PPP and as a byproduct, less production of NADPH, as shown in Figure 2A. While we also see only a modest increase in IMP and hypoxanthine in BJ fibroblasts, we see an increase in adenosine as well, which may suggest that de novo purine synthesis might be up-regulated because of CBD treatment. This contrasts with the decrease in de novo purine synthesis we see in Malme cells.

## 4. Discussion

**Figure 3:**
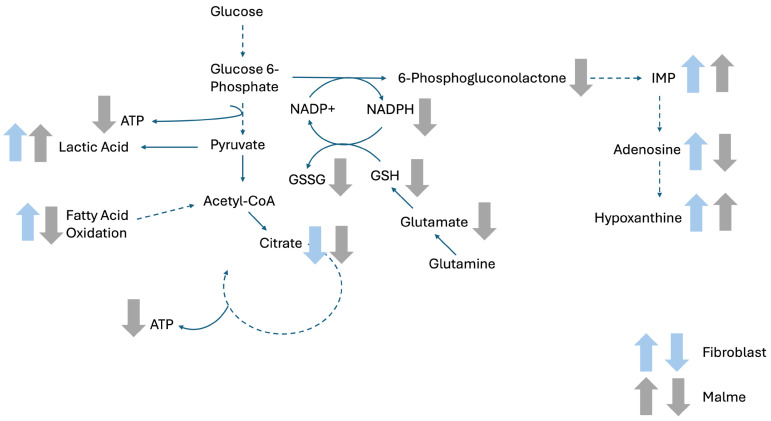
Pathway representation of the interconnected and differentially affected pathways as a result of CBD treatment. As shown, there was a clear differential response in the Malme cell line, where there was a clear reduction in ATP, glutamate, GSH, GSSG, adenosine, and fatty acids. In contrast, while the fibroblast cell line did experience an overall reduction in citrate with a corresponding elevation of lactic acid, there was no additional reduction in the glutathione related metabolites. In contrast to the Malme cell line, we saw an overall elevation of fatty acid metabolites, suggesting a metabolic switch to fatty acid oxidation to compensate for reduced citrate. As a result, the fibroblast cell line was able to maintain ATP levels, while the more-aggressive Malme cell line could not. Dashed arrows represent metabolic pathways with multiple intermediates not visible; full arrows show direct pathways with immediate precursors and products shown.

A detailed metabolic analysis of less-aggressive human BJ fibroblasts versus the malignant Malme-3M cell line was performed after 72 h of CBD treatment. CBD exerted a differential effect in the Malme-3M cell line, particularly affecting central energy metabolites, as highlighted in Figure 3. Specifically, there was a reduction of TCA cycle metabolites in the Malme-3M cells, and a corresponding increase in glycolytic output in the form of lactic acid. Consistent with an inhibited TCA cycle, reductions in ATP levels were seen. A potential consequence of an elevated glycolytic pathway is the diversion of glucose carbons away from de novo purine synthesis for the continued production of ATP for cell survival. However, because of reduced purine synthesis, the nucleotide salvage pathway appears to be elevated, as noted with dramatic increases in both hypoxanthine and IMP, key biomarkers of nucleotide salvage, in CBD treated Malme-3M cells. A major consequence of reduced carbon flow through the pentose phosphate pathway (PPP)—a key precursor pathway for de novo purine synthesis—is the reduction of NADPH, which is generated by the first committed step of the PPP. NADPH is critical for the recycling of oxidized glutathione during times of oxidative stress. Overall, in conjunction with a reduction of total glutathione levels in the Malme-3M cells, we suggest that this can further sensitize the cells to oxidative stress.

The reduction of total glutathione levels could be in part due to a reduction in de novo synthesis of glutathione as glutamate, a key glutathione precursor, could be re-directed to the TCA to compensate for the reduced TCA activity. A reduction in glutathione, in combination with reduced levels of NADPH, may result in an increased sensitivity to oxidative stress in the Malme-3M cells. Therefore, CBD treatment may result in synergistic effects when combined with other redox-inducing stress such as radiation therapy.

Interestingly, while the BJ fibroblasts also exhibited changes in some of the same key metabolic pathways, namely the TCA cycle and an increase in lactic acid production, there were key differences in the response by these cells. One key difference in the response to CBD treatment was the overall increase in short and medium chain fatty acids in the BJ cells, while either no change or a slight decrease in the Malme-3M cell line was seen. This differential response could be observed because of the inhibited TCA cycle. The less-aggressive cells may switch to fatty acid oxidation to maintain cellular ATP levels. Indeed, there was no change in ATP levels in the BJ cells, even in the presence of reduced citrate concentrations and a presumably reduced TCA cycle. On the other hand, Malme-3M cells were unable to maintain cellular ATP levels in a similar way. Instead, the attempt to maintain ATP levels was through an increase in glycolytic output, as evidenced by a larger increase in lactic acid in the Malme-3M cells.

The observed metabolic changes due to the mitochondria-related consequences of CBD treatment may be related to calcium maintenance. CBD has previously been shown to increase intracellular calcium levels and reduce cell viability [25,26]. These effects can be attributed to activation of the transmembrane ion channel TRPA1 by CBD, followed by activation of the mitochondrial outer membrane VDAC1 by increased calcium flux or direct interaction with CBD [27]. VDAC1 is an outer mitochondrial receptor that increases uptake of calcium into the mitochondria, leading to calcium overload and cell death. Studies have shown alterations of cancer cell mitochondrial membrane composition [28,29] could leave cancer cells more susceptible to calcium dysregulation because CBD exerts its effects through several potential signaling pathways. This can occur through the CB1/CB2, VDAC1, and TRPV1 receptors, which all regulate glycolysis, glutaminolysis, and oxidative stress markers. It is possible that future therapeutic strategies can take advantage of this differential response. For example, aggressive cancers treated with CBD may be more susceptible to therapies designed to increase oxidative stress, or other metabolic inhibitors used to synergistically weaken aggressive cancer cells.

## 5. Conclusions

For the first time, an analysis of metabolomics of both less-aggressive BJ fibroblasts and malignant Malme-3M cells after pre-treatment with CBD has been made. As a result of CBD treatment, there is reprogramming of key metabolic processes including the TCA cycle, redox maintenance, and fatty acid oxidation. While more studies are needed to fully elucidate the mechanistic effects of CBD treatment, there is a clear and differential cellular metabolic consequence of CBD treatment between these cell types.

## Figures and Tables

**Table 1:** Overall view of key metabolic changes observed in the Malme-3M cell line relative to the control BJ Fibroblasts. As shown in the table, several metabolites involved in primary metabolism, including choline regulation, glycolysis, and the TCA, were differentially regulated by CBD in the Malme cell line. The up-regulated cutoff, shown in red, was 1.2-fold or above, while the down-regulated cutoff, shown in green, was 0.8-fold or below. FC represents the fold change between the corresponding treated vs. control for the represented cell line. Black rows represent separators between major metabolic groups.

Annotation	FC Fibro CBD/Control	FC Malme CBD/Control	Pathway
Malonyl CoA	1.39	0.00	Fatty acid synthesis
HMG CoA	0.22	0.38	CHOL synthesis
			
Glucose 6-phosphate	0.54	0.46	Glycolysis
Fructose 6-phosphate	0.47	0.36	Glycolysis
Fructose 1,6-diphosphate	1.89	0.48	Glycolysis
Dihydroxyacetone phosphate (DHAP)	1.52	0.43	Glycolysis
2-Phosphoglyceric acid	0.75	0.59	Glycolysis
3-Phosphoglyceric acid	0.84	0.59	Glycolysis
Phosphoenolpyruvic acid (PEP)	0.70	0.38	Glycolysis
Pyruvic acid		0.86	Glycolysis
Lactic acid	1.80	1.67	Glycolysis
Lac/Pyr ratio		1.97	Glycolysis
*O*-Acetylcarnitine *(ALCAR)*	1.35	0.42	Glycolysis
Acetyl CoA	2.95	1.18	Glycolysis
ALCAR/Acetyl CoA	0.40	0.34	Glycolysis
3-Hydroxybutyric acid (3-HB)	1.21	0.61	Glycolysis
			
Met	1.02	1.00	Transulfuration
Cys	1.71	0.83	Transulfuration
Homocysteine	0.34	0.42	Transulfuration
*S*-Adenosylhomocysteine	1.24	0.91	Transulfuration
*S*-Adenosylmethionine	1.00	2.19	Transulfuration
Glutathione (GSSG)	0.99	0.43	Oxidative stress
Glutathione (GSH)	0.59	0.41	Oxidative stress
GSH/GSSG ratio	0.61	0.96	Oxidative stress
Cystathionine	1.60	0.99	Oxidative stress
Carnosine	1.07	0.60	Oxidative stress
NADH	1.72	0.29	NAD metabolism
NADPH	1.39	0.18	NAD metabolism
NADH/NADPH ratio	1.42	1.63	NAD metabolism
			
Phosphocreatine	0.35	0.02	Urea cycle
Ornithine	0.90	1.31	Urea cycle
Arg	1.49	1.26	Urea cycle
Citrulline	1.18	1.07	Urea cycle
Arg/Cit ratio	1.27	1.17	Urea cycle
Creatinine	1.11	0.92	Urea cycle
Creatine	0.52	0.31	Urea cycle
Spermine	0.72	0.28	polyamine
Spermidine	1.08	0.11	polyamine
*N*^1^,*N*^12^-Diacetylspermine		100.63	Polyamine
*N*^1^-Acetylspermine		5.28	Polyamine
*N*-Acetylputrescine	0.81	1.46	Polyamine
*N*^1^,*N*^8^-Diacetylspermidine		4.33	Polyamine
*N*^1^-Acetylspermidine	1.92	3.28	Polyamine
Putrescine	1.05	2.07	polyamine
*N*-Methylputrescine		1.47	polyamine
			
Inosine	3.65	0.34	purine
Adenosine	1.96	0.20	Purine synthesis
Guanosine	8.44	0.20	Purine synthesis
6-Phosphogluconic acid	0.89	0.68	Purine synthesis
Hypoxanthine	3.22	53.49	Purine synthesis
IMP	1.61	23.47	Purine synthesis
			
Citric acid	0.46	0.09	TCA cycle
*cis*-Aconitic acid	0.33	0.08	TCA cycle
Isocitric acid	0.00	0.00	TCA cycle
2-Oxoglutaric acid	0.56	0.03	TCA cycle
Succinic acid	1.15	7.39	TCA cycle, pyrimidine synthesis, urea cycle
Glutamic acid	0.93	0.17	TCA, Glutaminolysis, GSH synthesis
Glutamine	2.02	1.03	TCA, Glutaminolysis
Glu/Gln ratio	0.70	0.17	TCA cycle
Fumaric acid	0.72	0.51	TCA cycle
Malic acid	0.72	0.55	TCA cycle
FMN	0.00	0.14	TCA cycle
ATP	0.87	0.44	TCA cycle

## Data Availability

The original contributions presented in this study are included in the article/Supplementary Materials. Further inquiries can be directed to the corresponding author.

## Supplementary Materials

The following supporting information can be downloaded at: https://www.mdpi.com/article/10.3390/cimb47110928/s1.
