# Supplementary material for: CBD Disrupts Malme-3M Cell Metabolism via Glycolytic Shift and Redox Imbalance
Source: Curr Issues Mol Biol. 2025 Nov 6;47(11):928. doi: 10.3390/cimb47110928 (PMC12651666; doi:10.3390/cimb47110928)
Supplement: Supplementary file 1 [file cimb-47-00928-s001.zip › cimb-3913016-supplementary.pdf]

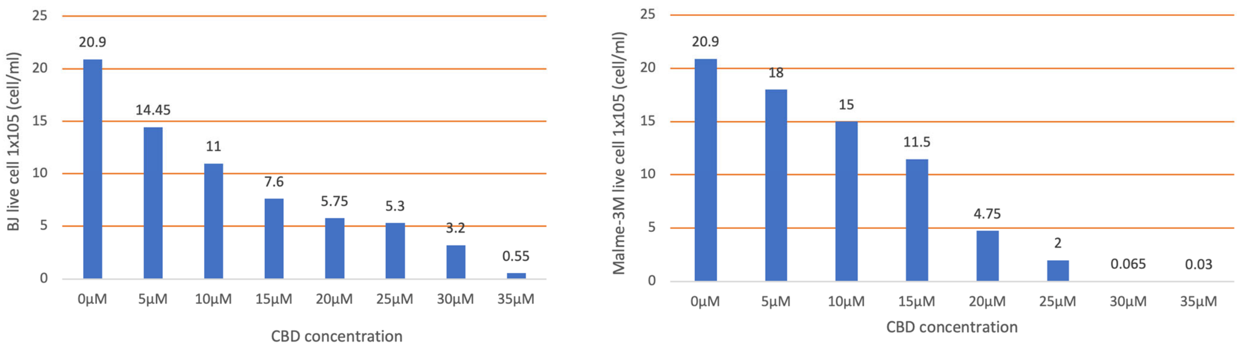

Supplementary Figure S1. BJ fibroblasts and Malme-3M cells were incubated for 72 hours with varying concentrations of CBD to determine the IC50 values. Viable cell counts are listed above each concentration marker. IC50 values were calculated to be 12.5 μM CBD for BJ Fibroblasts and 16μM CBD for Malme-3M.
